# Coronavirus disease 2019 and malaria coinfection in a middle-aged Ethiopian woman presenting with acute febrile illness and bilateral pleural effusion: a case report

**DOI:** 10.1186/s13256-023-04134-2

**Published:** 2023-08-31

**Authors:** Gashaw Solela, Addis Aschenek, Mikale Dawit, Getachew Wondafrash

**Affiliations:** 1 Department of Internal Medicine, Yekatit 12 Hospital Medical College, Addis Ababa, Ethiopia; 2 Division of Nephrology, Department of Internal Medicine, Yekatit 12 Hospital Medical College, Addis Ababa, Ethiopia

**Keywords:** COVID-19, Malaria, Pleural effusion

## Abstract

**Introduction:**

There could be misdiagnosis of coronavirus disease 2019 for malaria and vice versa because of their similar presentations, particularly when clinicians rely mainly on symptoms for diagnosis. Coinfection with coronavirus disease 2019 and malaria is associated with increased all-cause in-hospital mortality compared with isolated infection with severe acute respiratory syndrome coronavirus 2. Presentation with pleural effusion adds another challenge in the diagnosis of coronavirus disease 2019.

**Case presentation:**

This is a 57-year-old black Ethiopian woman who presented with symptoms of acute febrile illness associated with shortness of breath and coughing. Physical examination was remarkable for fever, hypotension, tachycardia, tachypnea, oxygen desaturation, decreased air entry, and dullness over bilateral lower one-third of the chest. Peripheral blood smear revealed ring-form trophozoites of *Plasmodium falciparum*; chest X-ray showed bilateral pleural effusion and chest computed tomography revealed bilateral ground-glass opacities and consolidations involving all lung zones with bilateral moderate pleural effusion. She was managed with supportive treatments, antimalarial agents, and antibiotics. Rapid antigen test for severe acute respiratory syndrome coronavirus 2 was negative at the time of her presentation to the emergency department, but polymerase chain reaction testing for coronavirus disease 2019 turned out to be positive after admission to the medical ward.

**Conclusion:**

Clinicians should be aware of the possibility of coronavirus disease 2019 and malaria coinfection in any patient who is from malaria-endemic area and presenting with acute febrile illness symptoms such as fever and headache and respiratory complaints like shortness of breath and cough. Alhough viral etiologies such as coronavirus disease 2019 are rare causes of bilateral pleural effusion, they should be considered after ruling out other common causes.

## Introduction

The World Health Organization (WHO) declared the outbreak of the novel coronavirus disease 2019 (COVID-19) global pandemic on 11 March 2020 [1]. Ethiopia reported the first COVID-19 case on 13 March 2020 [2]. COVID-19 has a wide range of manifestations, including asymptomatic stages and acute febrile illness symptoms such as fever, chills and myalgia, or pneumonia that may be complicated with respiratory failure and require inpatient care [3]. Although pleural effusion in severe acute respiratory syndrome coronavirus 2 (SARS-CoV-2) pneumonia is a less prevalent symptom, it has been seen more frequently than in other viral pneumonias [4].

SARS‐CoV‐2 and the resulting COVID‐19 pandemic present important diagnostic challenges. The COVID-19 pandemic could be controlled with the aid of accurate quick diagnostic tests for SARS-CoV2 infection. According to a systemic review of 155 cohort studies, rapid antigen tests have a sensitivity of 73% in symptomatic and 55% in asymptomatic patients with COVID-19, and the sensitivity was higher in the first week after symptom onset (80.9%) than in the second week (53.8%) [5]. Real-time reverse transcription polymerase chain reaction (RT-PCR) is a simple qualitative assay with a better sensitivity, but with similar specificity, compared to antigen tests used to diagnose COVID-19 infection [6, 7].

While COVID-19 and malaria can present similarly, they often share symptoms, such as fever, fatigue, and acute headaches, which can lead to a misdiagnosis of COVID-19 as malaria or vice versa [8]. This is especially true when a clinician focuses primarily on symptoms. Compared with SARS-CoV-2 infection alone, COVID-19 and malaria coinfection is linked to higher all-cause in-hospital mortality rates [9]. We present a case of COVID 19 and *Plasmodium falciparum* coinfection in a middle aged woman from Ethiopia who has presented with acute febrile illness and bilateral pleural effusion.

## Case presentation

This is a 57-year-old black Ethiopian female patient who presented to the emergency department (ED) of Yekatit 12 Hospital Medical College with fever, chills, and headache of a week duration associated with fatigue, shortness of breath, and dry cough. Three days prior to her presentation, she started to develop diarrhea, which subsided after her visit to our hospital. She had recent travel history to a malaria-endemic area. She received two doses (administered 3 months apart) of AstraZeneca COVID-19 vaccine 2 years prior to her current presentation, but she did not receive any booster dose. Upon physical examination she had fever, hypotension, tachycardia, tachypnea, oxygen desaturation to the level of 70% with room air, and decreased air entry and dullness over the lower one-third of the chest bilaterally.

**Fig. 1:**
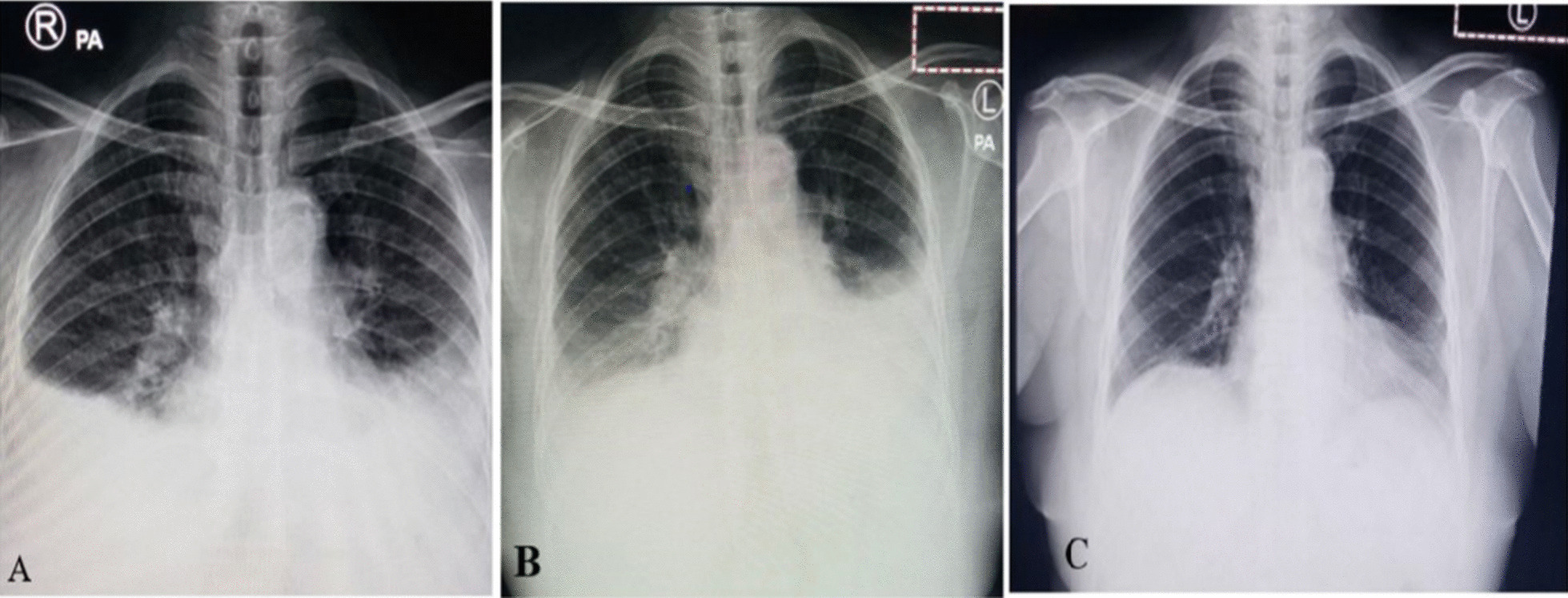
Serial chest X-ray images. **A** Symmetrical bilateral pleural effusion; **B** bilateral pleural effusion greater on the left side; **C** significant improvement, except for left basal haziness

On laboratory testing, complete blood count revealed mild lymphopenia (1000 cells/µl); peripheral blood smear revealed ring-form trophozoites of *Plasmodium falciparum*; renal and liver function tests were within normal ranges; initial pleural fluid analysis was serous with total cell count of 50 cells/µl, which increased to 3000 cells/µl (lymphocyte of 70%) with serous appearance, and non-remarkable protein, glucose, and LDH levels; erythrocyte sedimentation rate (ESR) was initially 146 mm/hour, which then dropped to 40 mm/hour on her 14th day of admission; and rapid antigen test for SARS-CoV-2 was negative at her presentation to the ED, but PCR testing for COVID-19 after admission to the medical ward turned out to be positive (Table 1). Upon imaging tests, serial chest X-rays (Fig. 1) showed symmetrical bilateral pleural effusion at presentation (Fig. 1A), left-side predominant bilateral pleural effusion on the 5th day of admission (Fig. 1B), and significant improvement on the 14th day of admission (Fig. 1C). Chest computed tomography (CT) with contrast revealed bilateral ground-glass opacities and consolidations involving all lung zones with bilateral moderate pleural effusions. Echocardiography revealed mild left ventricular hypertrophy (LVH) with grade I diastolic dysfunction.

**Table 1:** Laboratory findings

Laboratory tests	Results		
	At admission	On 5th day of admission	On 14th day of admission
Complete blood count			
White blood cell count (cells/µl)	6.8 × 10^3^	6.3 × 10^3^	
Neutrophil (%)	73	81	
Lymphocyte (%)	15	16	
Hemoglobin (g/dl)	12.7	12.9	
Platelet (cells/µl)	61 × 10^3^	203 × 10^3^	
Pleural fluid analysis			
Cell count (cells/µl)	50	3000	
Neutrophil (%)	75	30	
Lymphocyte (%)	25	70	
Protein (g/dl)		3.1	
LDH (U/L)		210	
Glucose (mg/dl)		160	
AFB		Negative	
Gene Xpert		Negative	
Gram stain		Negative	
Peripheral blood smear	Ring-form trophozoites of *P. falciparum*		
ESR (mm/hour)	Not done	146	40
SARS-CoV-2 RDT	Negative		
SARS-CoV-2 PCR			Positive*
HbA1c (%)		7.2	
Serum LDH (U/L)		520	
Creatinine (mg/dl)	0.7		

AFB, acid-fast bacillus; ESR, erythrocyte sedimentation rate; LDH, lactate dehydrogenase; RDT, rapid diagnostic test.*Arrival of the result was 9 days after sample collection

At the time of her initial presentation to ED, considering severe malaria with hypotension and bilateral pleural effusion with an unknown etiology, she was put on intranasal oxygen, resuscitated with intravenous fluid and was started on intravenous artesunate. After admission to the medical ward, intravenous artesunate was changed to oral arthemeter–lumefantrine; ceftriaxone with azithromycin and diuresis were initiated for consideration of both pneumonia with parapneumonic effusion and heart failure respectively.

Despite optimal diuresis and the aforementioned antibiotics, her respiratory condition was not improving and for this reason, antibiotics were revised to cefepime and vancomycin and chest CT angiography was also done suggesting multifocal pneumonia with bilateral parapneumonic effusion. On the 5th day of ward admission, control chest X-ray showed bilateral pleural effusion with left side predominance (Fig. 1B); therapeutic pleural tap was done on the left side with drainage of 500 ml, repeat pleural fluid analysis showed increased cell count (3000 cells/µl) with lymphocytic predominance (70%), and pleural fluid cytology showed no malignant cells. All the parameters in the repeated pleural fluid analysis were non-remarkable, except the increased cell count (Table 1). After reevaluation of the patient, viral cause like COVID-19 was entertained again and repeated testing with SARS-CoV-2 PCR was done and turned out to be positive, although the result arrived late (after 9 days of request). She was also screened for diabetes and her HbA1c was found to be 7.2%.

On the 10th day of patient’s admission, her condition began to improve, accompanied by a decreased oxygen requirement and a drop in ESR to 116 mm/hour. On the 14th day of her admission, ESR decreased further to 40 mm/hour and follow-up chest X-ray showed a significant radiologic improvement (Fig. 1C). Her final diagnosis was newly diagnosed type 2 diabetes mellitus, *Plasmodium falciparum* malaria, and COVID-19 pneumonia with parapneumonic effusion. She was discharged with marked improvement and upon serial evaluations at the outpatient department on her 1st and 3rd week of discharge, her symptoms had disappeared except for mild fatigue and physical examination was also non-remarkable.

## Discussion

In the early stages of the COVID-19 pandemic, endemic malaria appeared to be adequate to protect populations from the outbreak, particularly in less developed nations [10, 11]. The low incidence of COVID-19 in malaria-endemic nations has been attributed to angiotensin-converting enzyme 2 (ACE2), hydroxychloroquine (HCQ) and chloroquine (CQ), interferons, and neutralizing antibodies [8].

Due to unstable malaria transmission patterns, Ethiopia is susceptible to concentrated and significant cyclic malaria epidemics [12]. With *Plasmodium falciparum* infection, which can lead to a number of complications, prompt and precise diagnosis is extremely important for managing malaria. When suspecting a malaria case, it is essential to have a history of travel from regions where malaria is endemic at least 2 weeks prior to the onset of typical malaria symptoms [11]. Our patient had travel history to a malaria-endemic area and her peripheral blood smear showed *Plasmodium falciparum*.

The diagnosis of COVID-19 was initially ruled out for our patient upon her presentation at the emergency department due to the negative rapid antigen test for SARS-CoV-2, positive blood smear for *Plasmodium falciparum*, and chest X-ray revealing bilateral pleural effusion, which did not support a COVID-19 diagnosis. However, after admission to the medical ward, her findings could not be explained by a plausible diagnosis and COVID-19 test was repeated with SARS-CoV-2 PCR and it turned out to be positive.

Due to the SARS-CoV-2 fast antigen test’s inferior diagnostic accuracy for COVID 19, relying solely on these tests could result in a sizable percentage of people being mistakenly identified as SARS-CoV-2 negative [13]. The use of RT-PCR to detect SARS-CoV-2 nucleic acid in sputum or throat swabs and lower respiratory tract secretions for patients with suspected SARS-CoV-2 infection appears necessary for diagnosis [14], in addition to clinical and/or radiological symptoms.

Our patient had bilateral pleural effusion masquerading as heart failure, similar to a patient from Pakistan who had rheumatic heart disease, COVID-19-associated pneumonia, and bilateral pleural effusion [15]. Although the bilateral pleural effusion in our patient appeared to be symmetrical on the first chest X-ray, mimicking a sign of heart failure, it then showed left side predominance on the 5th day of admission, which did not correlate with a diagnosis of heart failure. According a systematic review of 23 studies, the average prevalence of pleural effusion in patients with COVID-19 is 9.55% and the presence of pleural effusion is associated with a higher severity of disease [16].

Although pleural effusion is rarely reported in patients with COVID-19 [17], viral etiologies like COVID-19 should be considered if alternative diagnoses are lacking. A narrative review on pleural abnormalities of confirmed cases of COVID-19 revealed that pleural fluid cellularity varied from 25 to 7738 cells/µl with lymphocyte predominance for the majority of the patients, LDH ranged from 79 to 3651 IU/L, protein ranged from 2 to 4.5 gm/dL, and glucose ranged from 102 to 1322 mg/dl. Most of the pleural fluid analysis results of our patient correlate with the findings of this review [18].

In a retrospective cohort study of 591 patients with a confirmed diagnosis of COVID-19, the coinfection rate with malaria was 45.7% and the majority of malaria patients (51.9%) were infected with *Plasmodium falciparum* [9]. COVID-19 cases with and without concurrent malaria had crude death rates of 10.71 and 5.87 per 1000 person-days in the same study, respectively [9]. Appropriate diagnostic investigations for both COVID-19 and malaria should be done for any patient presenting with respiratory complaints and symptoms of acute febrile illness in malaria-endemic areas.

## Conclusions

Clinicians should be aware of the possibility of COVID-19 and malaria coinfection in any patient who is from malaria-endemic area and presenting with acute febrile illness symptoms, such as fever and headache and respiratory complaints such as shortness of breath and cough. Although viral etiologies like COVID-19 are rare causes of bilateral pleural effusion, they should be considered after ruling out other common causes.

## Data Availability

Data supporting this case report will be available from the corresponding author on reasonable request.
